# Validation of a 5-item tool to measure patient assessment of clinician compassion in the emergency department

**DOI:** 10.1186/s12873-019-0279-5

**Published:** 2019-11-04

**Authors:** Praveen Sabapathi, Michael B. Roberts, Brian M. Fuller, Michael A. Puskarich, Christopher W. Jones, J. Hope Kilgannon, Valerie Braz, Christina Creel-Bulos, Nathaniel Scott, Kristina L. Tester, Anthony Mazzarelli, Stephen Trzeciak, Brian W. Roberts

**Affiliations:** 1 Department of Emergency Medicine, Cooper University Health Care, Cooper Medical School of Rowan University, One Cooper Plaza, K152, Camden, New Jersey 08103 USA; 2 Institutional Research and Outcomes Assessment, Philadelphia College of Osteopathic Medicine, Philadelphia, PA USA; 3 Department of Emergency Medicine, Washington University School of Medicine, St. Louis, MO USA; 4 Department of Anesthesiology, Division of Critical Care Medicine, Washington University School of Medicine, St. Louis, MO USA; 5 Department of Emergency Medicine, Hennepin County Medical Center, University of Minnesota, Minneapolis, MN USA; 6 Center for Humanism, Cooper Medical School of Rowan University, Camden, NJ USA; 7 School of Medicine, University of Minnesota, Minneapolis, MN USA

**Keywords:** Compassion, Empathy, Emergency department

## Abstract

**Background:**

To test if the 5-item compassion measure (a tool previously validated in the outpatient setting to measure patient assessment of clinician compassion) is a valid and reliable tool to quantify a distinct construct (i.e. clinical compassion) among patients evaluated in the emergency department (ED).

**Methods:**

Cross-sectional study conducted in three academic emergency departments in the U.S. between November 2018 and April 2019. We enrolled adult patients who were evaluated in the EDs of the participating institutions and administered the 5-item compassion measure after completion of care in the ED. Validity testing was performed using confirmatory factor analysis. Cronbach’s alpha was used to test reliability. Convergent validity with patient assessment of overall satisfaction questions was tested using Spearman correlation coefficients and we tested if the 5-item compassion measure assessed a construct distinct from overall patient satisfaction using confirmatory factor analysis.

**Results:**

We analyzed 866 patient responses. Confirmatory factor analysis found all five items loaded well on a single construct and our model was found to have good fit. Reliability was excellent (Cronbach’s alpha = 0.93) among the entire cohort. These results remained consistent on sub-analyses stratified by individual institutions. The 5-item compassion measure had moderate correlation with overall patient satisfaction (r = 0.66) and patient recommendation of the ED to friends and family (r = 0.57), but reflected a patient experience domain (i.e. compassionate care) distinctly different from patient satisfaction.

**Conclusions:**

The 5-item compassion measure is a valid and reliable tool to measure patient assessment of clinical compassion in the ED.

## Introduction

Compassion has been defined as the emotional response to another’s pain or suffering involving an authentic desire to help [1–3]. Although closely related to empathy, defined as the ability to understand another’s emotions, compassion is the responsive action that flows from that understanding and thus can be perceived by patients [1–5]. Clinician compassion is considered a vital aspect of high quality healthcare by patients and clinicians [6]. Not only is clinician compassion desired by patients, but it is also associated with improvement in clinical outcomes [7]. For example, compassionate care has been demonstrated to reduce patient fear and anxiety during medical care [8], and conversely, a lack of compassionate care in the emergency department (ED) is a cause of acute patient distress [9]. Further, a recent study found patient perception of greater clinical compassion in the ED during resuscitation of a potentially life threatening medical emergency to be associated with less post-traumatic stress disorder symptoms at 30 days after discharge [4]. Compassion is also vital for clinicians as current evidence suggests that increased clinician compassion is associated with more resilience, an improved state of well-being, and decreased rates of burnout [10–12]. This relationship between compassion and burnout may be especially important for emergency medicine clinicians who have the highest rates of burnout among medical specialties and a burnout prevalence that continues to rise [13]. Therefore the ED may be a unique context where increased compassion could provide maximal benefit for both patients and clinicians.

Given that compassionate care is associated with important patient- and clinician-oriented outcomes, having the ability to measure patient assessment of compassion (as opposed to clinician self-assessment of compassion or third party observation of compassion) in the ED would be an important advancement for assessing healthcare quality. However, a recent systematic review identified a need for a psychometrically validated instrument that comprehensively measures the construct of compassion in healthcare settings [5]. Realizing this need, we previously developed and validated a 5-item compassion measure for administration with the Clinician and Group Consumer Assessment of Healthcare Providers and Systems (CG-CAHPS) survey, a patient satisfaction survey for adult outpatient clinic visits used by the United States (U.S.) Centers for Medicare and Medicaid Services for all healthcare organizations that receive payments from Medicare [14]. Although we found the 5-item compassion measure to be a reliable tool to measure patient perception of clinician compassion in the outpatient setting, this tool has not yet been validated for use in the ED setting. Given the inherit differences in patient experiences during outpatient clinic visits compared to ED visits, the tool must be psychometrically validated among ED patients before it can be reliably used in the ED setting. The objective of this study was to validate the 5-item compassion measure for use in the ED. We hypothesized that the 5-item compassion measure is a valid and reliable tool to quantify a distinct construct (i.e. compassion) among patients evaluated in the ED.

## Methods

### Setting

This cross-sectional study was conducted in three academic emergency departments in the U.S. (Cooper University Hospital, Camden, New Jersey; Washington University in St. Louis, MO; and Hennepin County Medical Center, Minneapolis, MN). The study took place from November 2018 to April 2019. This study was approved by the institutional review board at each participating institution and is reported in accordance with the Strengthening the Reporting of Observational Studies in Epidemiology (STROBE) Statement for cross-sectional studies (Additional file 1) [15].

### 5-item compassion measurement tool

The 5-item compassion measure (Table 1) was previously developed and prospectively validated among patients who had an outpatient clinic visit across 15 different specialties. Our previous results found the 5-item compassion measure to be a valid and reliable tool to measure patient perception of clinician compassion on a large scale in the outpatient setting. The results of the pilot and validation studies were previously published [14]. Further, the 5-item compassion measure is easy to read with a Flesch–Kincaid grade level (an established method for providing reliable and reproducible scores of readability) of 6.4 [16, 17].

**Table 1:** 5-item compassion measure and patient satisfaction items

5-item compassion measure* During this emergency department visit, how often do you feel your clinician...	
1. Cared about your emotional or psychological well-being?	
2. Was interested in you as a whole person?	
3. Was considerate of your personal needs?	
4. Was able to gain your trust?	
5. Showed you care and compassion?	

* Each item response scaled as 1 = never; 2 = sometimes; 3 = usually; 4 = always

### Study population and survey administration

We enrolled a convenience sample of patients age ≥ 18, who were evaluated in the EDs of the participating institutions, and were capable of answering the survey questions (i.e. English speaking and had capacity to read and answer questions). When available, volunteer research assistants proffered patients the research survey for completion, which included the 5-item compassion measure (Table 1) and two questions about patient satisfaction that were adapted from the CG-CAHPS survey [18]: (1) using any number from 0 to 10, where 0 is the worst care possible and 10 is the best care possible, what number would you use to rate your care during this emergency room visit? and (2) would you recommend this emergency room to your friends and family? (1, Definitely no; 2, Probably no; 3, Probably yes; or 4, Definitely yes). Surveys were administered at the time ED clinician care was completed (i.e. the time the discharge order or admission order was placed by the ED clinician) and were returned to the research assistants prior to patients leaving the ED. Research assistants were typically in the ED between 8:30 am and 10:30 pm, seven days per week and were trained to administer the research survey at the completion of patient care in the ED (i.e. at the time of discharge to home or admission to the hospital). In an effort to minimize the risk of response bias, no patient identifiers were recorded and patients were informed that their clinician would not have access to the survey results. Data were entered into Research Electronic Data Capture (REDCap), a secure, web-based application designed to support data capture for research studies [19], and exported into Stata/SE 15.1 for Mac, StataCorp LP (College Station, TX, USA) for analysis.

### Statistical analysis

Patient survey responses were described using median and interquartile range, or mean and standard deviation for continuous variables, and frequency and proportions for categorical variables. We calculated the complete response rate for the 5-item compassion measure among surveys administered.

Confirmatory factor analysis (using structural equation modeling) was used on the 5-item compassion measure to evaluate how correctly the hypothesized model (in this case a theorized single construct) matched the observed data, as well as to calculate standardized coefficients for each item. Given the non-normality of the data (i.e. ordinal data) we used the Satorra and Bentler scaled chi-squared test, which provides a scaled version of the chi-squared statistic that more closely follows the mean of the reference distribution in the presence of non-normally distributed data [20]. As previously performed, we examined fit indices (which take into account total sample size), including Comparative Fit Index (CFI), Tucker-Lewis Index (TLI), and standardized root mean squared residual (SRMR). We a priori chose our model to have good fit if CFI > 0.95, TLI > 0.95, and SRMR < 0.08 [14, 21]. We chose to examine fit indices because, when the sample size is large, the chi-square test for model fit is often significant (i.e. suggesting model is a poor fit) even when the model is, in practice, a good fit [14, 22, 23]. We then performed the same analysis stratified by institution to ensure the model had good fit across the different EDs.

Reliability was tested among the entire cohort, as well as among each individual institution, using Cronbach’s alpha. We summed the scores for each individual item to obtain a composite score for the 5-item compassion measure. Using Spearman correlation coefficients we tested convergence validity between the 5-item compassion measure total score and the two items assessing patient satisfaction with care. We hypothesized that the 5-item compassion measure would have a positive correlation with, yet be distinct from, the patient satisfaction questions. To further test if the items in the 5-item compassion measure form a discrete construct (and do not simply reflect patient satisfaction) we tested the null hypothesis that the covariance between the two latent structures is 1 (i.e. single construct model) [14]. To test this hypothesis we used a likelihood ratio test to compare two nested models: one model with covariance between the two latent models constrained at 1 (i.e. single construct) vs. a second model with covariance between the two latent models allowed to be a free parameter (i.e. two construct model) [14]. We also report the fit indices for the two-construct model and used the a priori definition for good fit described above.

## Results

**Fig. 1:**
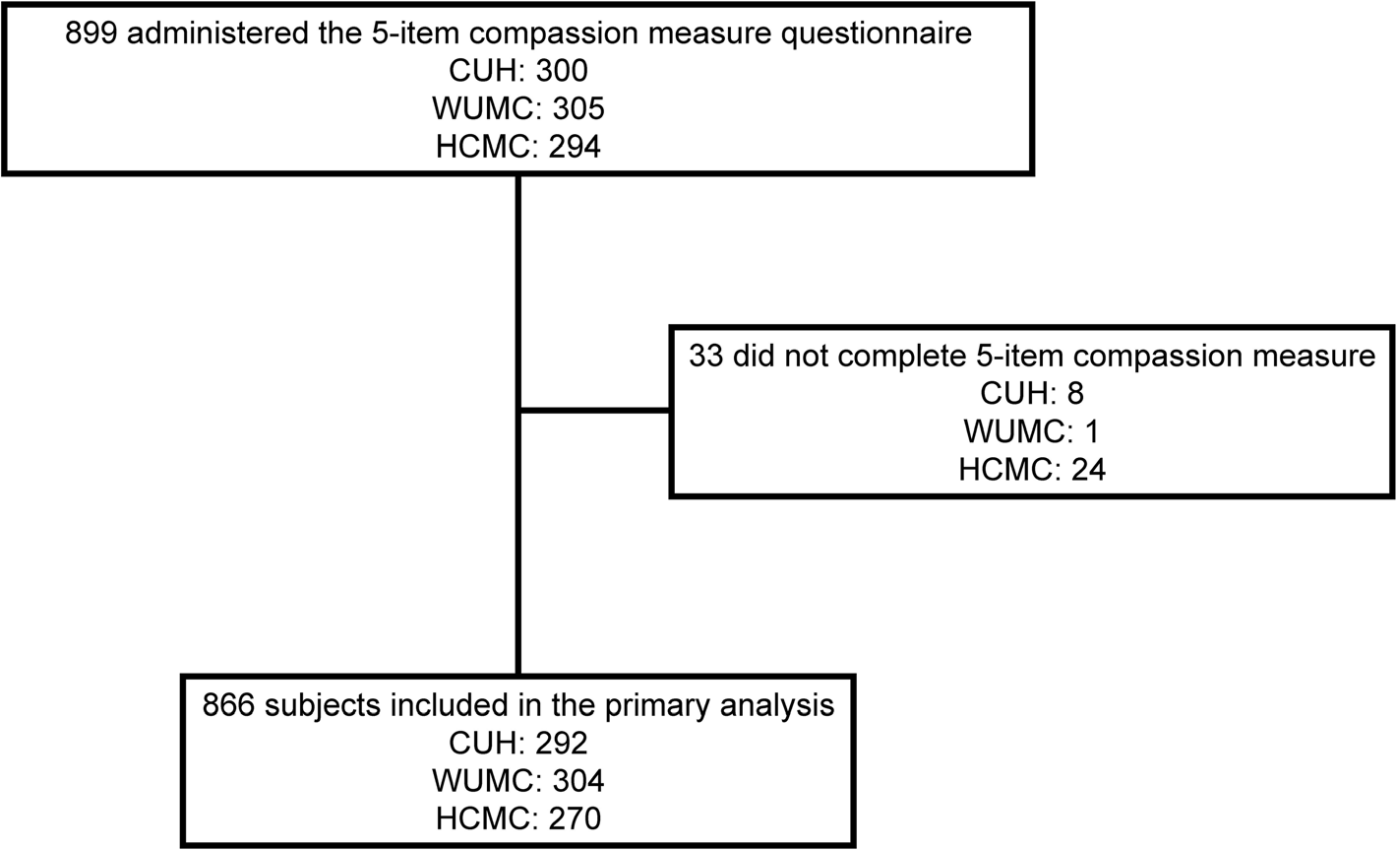
Study flow diagram. CUH, Cooper University Hospital; WUMC, Washington University in St. Louis Medical Center; HCMC, Hennepin County Medical Center

Response rates are displayed in Fig. 1. Patient self-reported characteristics are displayed in Table 2. Patient age ranged from 18 to 93 years. Fifty-five percent of participants were female, and 40% had some degree of college education. The cohort was diverse with respect to race and ethnicity. Fifty-two percent of patients were discharged home from the ED.

**Table 2:** Patient characteristics

Variable	Cohort (*n* = 866)
Age [mean (SD)]	47 (18)
Female [*n* (%)]	475 (55)
Race [*n* (%)]	
White	328 (38)
Black/African American	444 (51)
Asian	14 (2)
Hawaiian/Other Pacific Islander	4 (0.5)
American Indian/Alaska Native	28 (3)
Other	72 (8)
Hispanic or Latino decent [*n* (%)]	90 (10)
Highest education level completed [*n* (%)]	
8th grade or less	39 (5)
Some high school	153 (18)
High school graduate	325 (38)
Some college	197 (23)
4 years college graduate	101 (12)
4+ years college	46 (5)
Unknown	5 (0.6)
Reason for ED visit [*n* (%)]	
Accident or injury	132 (15)
New health problem	383 (44)
Ongoing health condition or concern	349 (40)
Discharged to home [*n* (%)]	454 (52)

*ED* emergency department, *SD* standard deviation

Confirmatory factor analysis found all five items loaded well on a single construct (Table 3). We found our model had good fit based on our a priori definition: CFI = 1, TLI = 0.99, and SRMR = 0.02. Given the large sample size, as expected the chi-square test for model fit was significant, *p* = 0.042. The model was found to have good fit across all three institutions: Cooper University Hospital (CFI = 0.99, TLI = 0.99, SRMR = 0.02, chi-square test *p* = 0.157), Washington University in St. Louis (CFI = 1, TLI = 1, SRMR = 0.01, chi-square test *p* = 0.721), and Hennepin County Medical Center (CFI = 0.99, TLI = 0.99, SRMR = 0.03, chi-square test *p* = 0.234).

**Table 3:** Standardized coefficients from confirmatory factor analysis

5-item compassion measure	Standardized coefficient
Cared about your emotional or psychological well-being?	0.78
Was interested in you as a whole person?	0.89
Was considerate of your personal needs?	0.89
Was able to gain your trust?	0.84
Showed you care and compassion?	0.83

Reliability was excellent (Cronbach’s alpha = 0.93) among the entire cohort, as well as across the three institutions: Cooper University Hospital (alpha = 0.93), Washington University in St. Louis (alpha = 0.95), and Hennepin County Medical Center (alpha = 0.89). The 5-item compassion measure ranged the full scale (5 to 20), and 49% of respondents gave perfect scores (i.e. score of 20). Additional file 1: Figures S1-S5 display the frequency for response scores for each individual item for the 5-item compassion measure. The 5-item compassion measure had a moderate correlation with overall patient satisfaction [r = 0.66 (95% CI 0.62–0.69)] and recommendation of the ED to friends and family [r = 0.57 (95% CI 0.52–0.61)]. Given the likelihood ratio test comparing the two nested models was statistically significant, we reject the null hypothesis that the covariance between the two latent structures is 1 (i.e. the two factor model has better fit). We found the two-construct model to have good fit, CFI = 0.99, TLI = 0.99, SRMR = 0.02, chi-square test *p* = 0.030. These results suggest that the items in the 5-item compassion measure quantify a discrete construct and do not simply reflect overall patient satisfaction with the ED visit.

## Discussion

This study provides validation of the 5-item compassion measure to assess patient perception of clinician compassion in the ED. To accomplish this aim, we enrolled a moderately large number of racially diverse emergency department patients both admitted to the hospital and discharged home in geographically distinct areas of the United States, while maintaining excellent response rates. We found the 5-item compassion measure to be valid and reliable across three academic institutions, as demonstrated by good model fit and the consistently high alpha across institutions. The 5-item compassion measure was found to have only a moderate correlation with patient satisfaction. This suggests the 5-item compassion measure is not another (redundant) patient satisfaction measure. In addition, confirmatory factor analysis found the 5-item compassion measure assesses a separate construct from patient satisfaction. These results have implications for both patients and clinicians.

There is currently evidence supporting an association between compassionate care and better clinical outcomes for patients [4, 7, 24–30]. Alternatively, a lack of compassion is associated with lower quality of care and increased risk of harm to patients through medical errors [31]. However, despite the substantial evidence demonstrating the importance of compassionate care, there is currently a lack of (or inconsistency in) compassionate care across health care systems globally [7], with physicians frequently overlooking opportunities to be compassionate, instead taking a narrow biomedical focus during bedside patient encounters [32]. Thus, having a means to assess patient perception of compassionate care in the ED is of the utmost importance.

Historically, it has been thought that investing oneself in patients may be emotionally demanding and could have a negative effect on clinician well-being (i.e. “empathic distress”) [3]. However, recent data suggest that clinician compassion can promote long-term resilience and well-being for clinicians and therefore may represent a method of counteracting or preventing burnout [10–12].

Emergency medicine is a very high stress discipline. Emergency medicine clinicians frequently have secondary exposure to trauma, which is now a valid Diagnostic and Statistical Manual of Mental Disorders (DSM)-5 criterion stressor for posttraumatic stress disorder [33]. Further, the prevalence of burnout among emergency medicine clinicians continues to rise, and emergency medicine clinicians have the highest rate of burnout across all medical specialties [13]. Identifying means to reduce stress and burnout, and improve job satisfaction is therefore paramount to emergency medicine clinicians.

Being compassionate is not simply an inherent trait, which clinicians either do or do not possess; rather, recent evidence supports that compassionate behaviors can be learned through training and practice [2, 34]. Thus, by being able to measure patient assessment of compassion in the ED it will be possible to identify physicians who could potentially benefit from interventions to promote compassionate care. In addition, further research is needed to develop and test if interventions aimed at promoting compassion among clinicians improves patient outcomes and/or decreases clinician burnout. Having the ability to measure patient assessment of compassion will be vital for such research studies.

We acknowledge that this study has important limitations to consider. First, this study was performed in three academic EDs, thus it is possible that a study performed among a different population would find different results. However, our consistent good model fit and reliability across all three sites provides evidence of generalizability. Second, due to staffing constraints we performed a convenience sample, as opposed to enrolling consecutive patients. Thus, we are unable to report the total number of patients who presented to the ED who met all inclusion and no exclusion criteria. However, we believe our sample is well representative of the ED population as a whole at our study sites given the inclusion of patients discharged to home as well as those admitted to the hospital, and the large, demographically diverse sample size across the three institutions. Furthermore, as the goal of this study was to validate the 5-item compassion measurement tool in the ED (i.e. not to measure or quantify compassion at this time), it is unlikely that having consecutive patients would have significantly altered our results. In addition, we administered the compassion measure while patients were still in the ED in order to avoid lower response rates typically associated with after-care mail surveys and the potential for non-response bias. Third, completion of the study questionnaire (i.e. study participation) was voluntary. Figure 1 displays the number of patients from each site who chose not to complete the questionnaire. It is unknown why subjects chose not to complete the questionnaire in the ED, specifically why a greater number of patients from Hennepin County Medical Center chose not to complete the questionnaire. However, the overall completion rate was 96%, and our psychometric results were similar between the three centers. Fourth, it is possible that patient assessment of clinician compassion is influenced not only by clinician behaviors, but also by clinician characteristics (e.g. clinician sex, age) and non-clinician variables (e.g. illness severity, ED length of stay). Further, it has previously been demonstrated that the ED environment, such as hallway care [35], perceptions of neighboring patients’ risk [36], and ED crowding [37], may impact psychological perceptions of care. Thus, further research is needed to determine what clinician behaviors (e.g. eye contact, statements of support), clinician characteristic, and non-clinical variables, if any, impact patient assessment of compassion. Specifically, the 5-item compassion measure could be used to identify potentially modifiable variables to improve patient perception of clinician compassion. Fifth, we only tested the psychometrics of the English version of the 5-item compassion measure and future research is needed to test the validity of the 5-item compassion measure in different languages. Sixth, this current study tested the psychometrics of the 5-item compassion measure at one point in time and future research is required to test if the 5-item compassion measure can be used to trend patient perception of compassion over time.

## Conclusion

In summary, the 5-item compassion measure appears to be a reliable tool to measure patient perception of clinician compassion in the ED. Future studies among differing cohorts are warranted to further test generalizability of this measurement tool. This provides a framework in which to measure clinical compassion as part of future trials testing interventions aimed at improving patient- and clinician-oriented outcomes.

## Additional file

**Additional file 1: Figure S1.** Distribution of the 5-item compassion measure question, “How often do you feel your clinician cared about your emotional or psychological well-being?”. **Figure S2.** Distribution of the 5-item compassion measure question, “How often do you feel your clinician was interested in you as a whole person?”. **Figure S3.** Distribution of the 5-item compassion measure question, “How often do you feel your clinician was considerate of your personal needs?”. **Figure S4.** Distribution of the 5-item compassion measure question, “How often do you feel your clinician was able to gain your trust?”. **Figure S5.** Distribution of the 5-item compassion measure question, “How often do you feel your clinician showed you care and compassion?”.

## Data Availability

The datasets used and/or analyzed during the current study are available from the corresponding author on reasonable request.

## Abbreviations

CFI: Comparative Fit Index
CG-CAHPS: Clinician and Group Consumer Assessment of Healthcare Providers and Systems
ED: Emergency department
SRMR: Standardized root mean squared residual
TLI: Tucker-Lewis Index
