# Supplementary material for: Validation of a 5-item tool to measure patient assessment of clinician compassion in the emergency department
Source: BMC Emerg Med. 2019 Nov 4;19:63. doi: 10.1186/s12873-019-0279-5 (PMC6827199; doi:10.1186/s12873-019-0279-5)
Supplement: Supplementary file 1 — Additional file 1: Figure S1. Distribution of the 5-item compassion measure question, “How often do you feel your clinician cared about your emotional or psychological well-being?”. Figure S2. Distribution of the 5-item compassion measure question, “How often do you feel your clinician was interested in you as a whole person?”. Figure S3. Distribution of the 5-item compassion measure question, “How often do you feel your clinician was considerate of your personal needs?”. Figure S4. Distribution of the 5-item compassion measure question, “How often do you feel your clinician was able to gain your trust?”. Figure S5. Distribution of the 5-item compassion measure question, “How often do you feel your clinician showed you care and compassion?”. [file 12873_2019_279_MOESM1_ESM.docx]

**Validation of a 5-item tool to measure patient assessment of clinician compassion in the emergency department**

**Supplemental Material**

Praveen Sabapathi, MD^1^; Michael B. Roberts, PsyD^2^; Brian M. Fuller, MD, MSCI^3,4^;

Michael A. Puskarich, MD^5,^; Christopher W. Jones, MD^1^; J. Hope Kilgannon, MD^1,7^;

Valerie Braz PhD^1^; Christina Creel-Bulos RN-BSN,MD^3^; Nathaniel Scott, MD^5^;

Kristina L. Tester, BA^6^; Anthony Mazzarelli, MD, JD, MBE^1,7^; Stephen Trzeciak, MD, MPH^7,8^;

Brian W. Roberts, MD, MSc^1,7^

1: Department of Emergency Medicine, Cooper University Health Care, Cooper Medical School of Rowan University, Camden, NJ

2: Institutional Research and Outcomes Assessment, Philadelphia College of Osteopathic Medicine, Philadelphia, PA

3: Department of Emergency Medicine, Washington University School of Medicine, St. Louis, Missouri

4: Department of Anesthesiology, Division of Critical Care Medicine, Washington University School of Medicine, St. Louis, Missouri

5: Department of Emergency Medicine, Hennepin County Medical Center, University of Minnesota, Minneapolis, MN

6: School of Medicine, University of Minnesota, Minneapolis, MN

7: Center for Humanism, Cooper Medical School of Rowan University, Camden, NJ

8: Department of Medicine, Cooper University Health Care, Cooper Medical School of Rowan University, Camden, NJ

For submission to *BMC Emergency Medicine*

Address for correspondence:

Brian Roberts, MD, MSc

Cooper University Hospital

One Cooper Plaza, K152

Camden, New Jersey 08103

Phone: (856) 342-2351

E-mail: roberts-brian-w@cooperhealth.edu

STROBE Statement—Checklist of items that should be included in reports of ***cross-sectional studies***

|  | Item No | Recommendation |
| --- | --- | --- |
| **Title and abstract** | 1 | (*a*) Indicate the study’s design with a commonly used term in the title or the abstract page 1 |
|  |  | (*b*) Provide in the abstract an informative and balanced summary of what was done and what was found page 1 |
| Introduction | | |
| Background/rationale | 2 | Explain the scientific background and rationale for the investigation being reported pages 3-4 |
| Objectives | 3 | State specific objectives, including any prespecified hypotheses page 4 |
| Methods | | |
| Study design | 4 | Present key elements of study design early in the paper page 5 |
| Setting | 5 | Describe the setting, locations, and relevant dates, including periods of recruitment, exposure, follow-up, and data collection page 5 |
| Participants | 6 | (*a*) Give the eligibility criteria, and the sources and methods of selection of participants pages 5-6 |
| Variables | 7 | Clearly define all outcomes, exposures, predictors, potential confounders, and effect modifiers. Give diagnostic criteria, if applicable pages 5-6 |
| Data sources/ measurement | 8* | For each variable of interest, give sources of data and details of methods of assessment (measurement). Describe comparability of assessment methods if there is more than one group pages 5-6 |
| Bias | 9 | Describe any efforts to address potential sources of bias pages 5-8 |
| Study size | 10 | Explain how the study size was arrived at N/A |
| Quantitative variables | 11 | Explain how quantitative variables were handled in the analyses. If applicable, describe which groupings were chosen and why pages 6-8 |
| Statistical methods | 12 | (*a*) Describe all statistical methods, including those used to control for confounding |
|  |  | (*b*) Describe any methods used to examine subgroups and interactions |
|  |  | (*c*) Explain how missing data were addressed |
|  |  | (*d*) If applicable, describe analytical methods taking account of sampling strategy |
|  |  | (*e*) Describe any sensitivity analyses pages 6-8 |
| Results | | |
| Participants | 13* | (a) Report numbers of individuals at each stage of study—eg numbers potentially eligible, examined for eligibility, confirmed eligible, included in the study, completing follow-up, and analysed. Figure 1 |
|  |  | (b) Give reasons for non-participation at each stage Figure 1 |
|  |  | (c) Consider use of a flow diagram Figure 1 |
| Descriptive data | 14* | (a) Give characteristics of study participants (eg demographic, clinical, social) and information on exposures and potential confounders. Table 2 |
|  |  | (b) Indicate number of participants with missing data for each variable of interest Table 2 |
| Outcome data | 15* | Report numbers of outcome events or summary measures page 9 |
| Main results | 16 | (*a*) Give unadjusted estimates and, if applicable, confounder-adjusted estimates and their precision (eg, 95% confidence interval). Make clear which confounders were adjusted for and why they were included N/A |
|  |  | (*b*) Report category boundaries when continuous variables were categorized N/A |
|  |  | (*c*) If relevant, consider translating estimates of relative risk into absolute risk for a meaningful time period N/A |
| Other analyses | 17 | Report other analyses done—eg analyses of subgroups and interactions, and sensitivity analyses Pages 9-10 |
| Discussion | | |
| Key results | 18 | Summarise key results with reference to study objectives Page 11 |
| Limitations | 19 | Discuss limitations of the study, taking into account sources of potential bias or imprecision. Discuss both direction and magnitude of any potential bias page 13 |
| Interpretation | 20 | Give a cautious overall interpretation of results considering objectives, limitations, multiplicity of analyses, results from similar studies, and other relevant evidence Pages 13-14 |
| Generalisability | 21 | Discuss the generalisability (external validity) of the study results Pages 13-14 |
| Other information | | |
| Funding | 22 | Give the source of funding and the role of the funders for the present study and, if applicable, for the original study on which the present article is based. No funding. |

*Give information separately for exposed and unexposed groups.

**Note:** An Explanation and Elaboration article discusses each checklist item and gives methodological background and published examples of transparent reporting. The STROBE checklist is best used in conjunction with this article (freely available on the Web sites of PLoS Medicine at http://www.plosmedicine.org/, Annals of Internal Medicine at http://www.annals.org/, and Epidemiology at http://www.epidem.com/). Information on the STROBE Initiative is available at www.strobe-statement.org.

**Supplemental Methods**

**Supplemental Figure 1**: Distribution of the 5-item compassion measure question, “How often do you feel your clinician cared about your emotional or psychological well-being?”

**Supplemental Figure 2**: Distribution of the 5-item compassion measure question, “How often do you feel your clinician was interested in you as a whole person?”

**Supplemental Figure 3**: Distribution of the 5-item compassion measure question, “How often do you feel your clinician was considerate of your personal needs?”

**Supplemental Figure 4**: Distribution of the 5-item compassion measure question, “How often do you feel your clinician was able to gain your trust?”

**Supplemental Figure 5**: Distribution of the 5-item compassion measure question, “How often do you feel your clinician showed you care and compassion?”
